# Frequently Relapsing Anterior Nodular Scleritis as the Initial Presentation of a Lethal Systemic Infection: Disseminated Tuberculosis with HIV Coinfection

**DOI:** 10.1155/2020/9020864

**Published:** 2020-01-31

**Authors:** A. G. T. A. Kariyawasam, C. L. Fonseka, P. U. T. De Silva, A. D. S. S. Sanjeewa, D. N. Wijewickrama, H. M. M. Herath, T. P. Weerarathna

**Affiliations:** ^1^University Medical Unit, Teaching Hospital Karapitiya, Galle, Sri Lanka; ^2^Department of Internal Medicine, Faculty of Medicine, University of Ruhuna, Galle, Sri Lanka; ^3^Teaching Hospital Karapitiya, Galle, Sri Lanka; ^4^Department of Venereology, Teaching Hospital Mahamodara, Galle, Sri Lanka

## Abstract

**Background:**

Scleritis is a painful inflammatory ocular disease often associated with an underlying systemic illness mostly having an autoimmune aetiology. Tuberculosis usually presents as pulmonary infection, and the ocular presentation is considered to be rare. *Case presentation*. We present a male who developed prolonged pyrexia following systemic steroids while being investigated for a frequently relapsing anterior scleritis. Biopsy of sclera demonstrated acid-fast bacilli, and histology of sclera and lymph node showed granulomatous inflammation with caseation. Contrast CT demonstrated mediastinal and visceral lymphadenopathy with pulmonary changes suggesting disseminated tuberculosis. Later, Western blot confirmed coinfection with HIV with a CD4 count of 71 cells/mm^3^. He was treated with antituberculous medications, and then HAART was initiated within two months. He showed good response and showed a partial resolution of scleritis at the end of two months.

**Conclusion:**

Tuberculosis tends to occur in unusual sites when coinfected with HIV. Scleritis is a rare extrapulmonary manifestation of tuberculosis. High degree of suspicion is critical in making diagnosis and commencing early treatment.

## 1. Introduction

Scleritis is a common disease in ophthalmology where more than 50% of the patients are found to have an underlying systemic illness [1–4]. The incidence of infectious aetiology in scleritis is considered to be less than 10% [1, 2, 4]. Tuberculosis represents only about 10.6% of all infectious scleritis [5]. Though rare, the infections should always be considered in cases of scleritis having a progressive course with suppurative necrosis and if the response to systemic immunosuppressive therapy is nonsatisfactory.

Tuberculosis, presenting as an ocular infection, is a rare form of extrapulmonary tuberculosis and is among the few systemic infections that can cause scleritis [1, 3, 5–7]. It is considered advisable to test for tuberculosis (TB) in tropical setting in patients presenting with scleritis where TB is considered to be endemic. Extrapulmonary TB is reported in 15 to 20 percent of all cases of TB in the general population [5–8], whereas this incidence rose to 50% in HIV-infected population [7]. Ocular TB is considered to be extremely rare with an incidence ranging from 1.4% to 18% in various studies [9–11]. HIV infection is one of the well-known risk factors for developing ocular TB [5, 10–13].

We present an unusual presentation of disseminated TB coinfected with HIV, with initial presentation with isolated recurring scleritis.

## 2. Case Report

**Figure 1:**
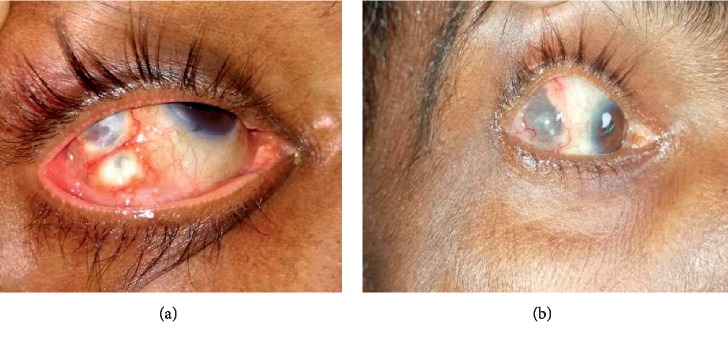
Anterior nodular scleritis of the right eye (a) before treatment with ATT and HAART and (b) after 2 months of treatment.

A 63-year-old man presented to the ophthalmologist with complaints of painful red eye on his right. His vision was satisfactory, and there were no complaints on the opposite eye. He denied any trauma or ocular surgery in the past. There were no systemic complaints such as joint pains, dryness of mouth, fever, cough, or any evidence of renal involvement. He was averagely built with normal systemic examination. Ophthalmological examination revealed bilateral normal visual acuity and normal intraocular pressure. The sclera of the right eye was inflamed with multiple nodules on the temporal sclera. The left eye was normal. He was diagnosed as having anterior nodular scleritis (Figure 1(a)) and was treated with topical steroids. Initial laboratory investigations were normal, and an apparent systemic cause for the scleritis could not be found. His chest radiograph, Mantoux test, sputum for AFB, and ultrasound scan of the abdomen were negative. The initial autoimmune markers (ANA, ANCA, and RF) were also negative.

During the next two months, he experienced frequent relapses of scleritis with gradual worsening with a poor response to the topical steroids so that the ophthalmologist decided to proceed with systemic steroids considering the progressive nature of the disease which appeared sight threatening. He received 3 pulses of intravenous methyl-prednisolone, but he did not have a durable response. Thereafter, the patient developed high-grade continuous fever for three weeks despite broad spectrum systemic antibiotics (intravenous ceftriaxone and doxycycline), and the blood, urine, and sputum cultures revealed negative results.

Thereafter, he was referred to medical ward for further evaluation of prolonged pyrexia. His investigations were full blood count showed lymphopenia with a high monocyte count (WBC: 5.9 × 10^3^/*μ*L, neutrophils: 4.47 × 10^3^/*μ*L (76%), lymphocytes: 0.83 × 10^3^/*μ*L (14%), and monocytes: 0.49 × 10^3^/*μ*L (8.2%)). There was a mild normochromic normocytic anaemia with a hemoglobin count of 8.8 g/dL, and the platelet count was normal (204 × 10^3^/*μ*L). His inflammatory markers were elevated (CRP 121 ⟶ 100 ⟶ 128), and the ESR was 79 ⟶ 100 ⟶ 134 mm/1^st^ hour). Repeat chest radiograph and ultrasound scan of the abdomen were normal.

**Figure 2:**
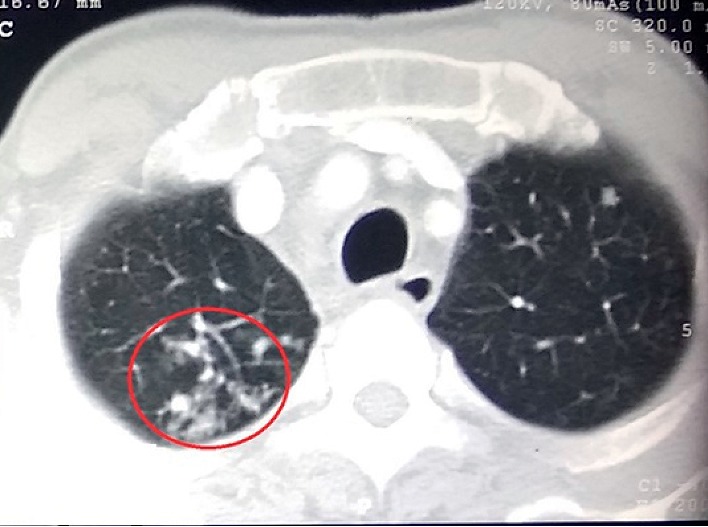
Contrast CT showing “tree-in-bud” appearance in the right upper lobe (circle) of the lung.

**Figure 3:**
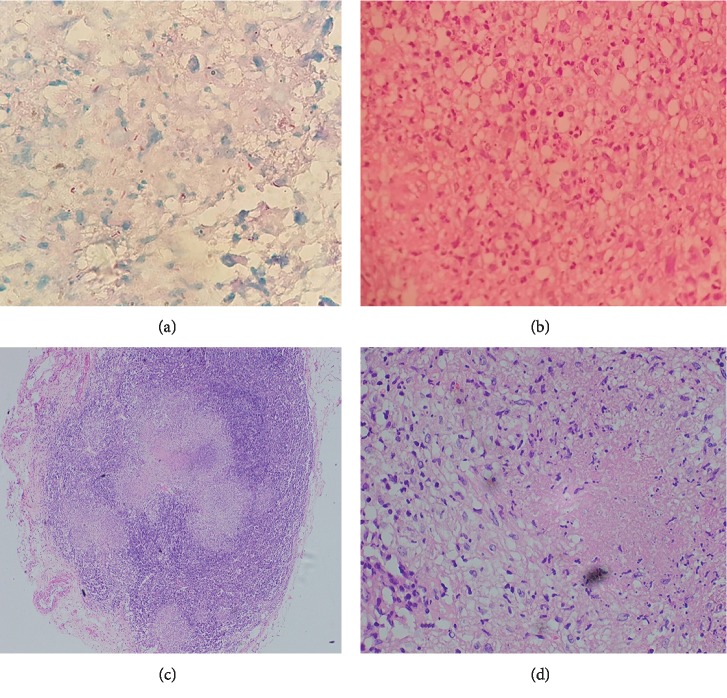
(a) Acid-fast bacilli in scleral tissue; (b) histology of the sclera showing granulomatous inflammation with caseation; (c) lymph node histology revealing granulomatous inflammation with caseation (low power); (d) lymph node histology (high power).

Re-examination disclosed the new occurrence of bilateral cervical and inguinal lymphadenopathy so that we proceeded for a cervical lymph node biopsy. The computed tomography of chest and abdomen revealed mediastinal, para-aortic, and inguinal lymphadenopathy with tree-in-bud pattern in the right lung apex suggesting the possibility of tuberculosis (Figure 2). By this time, the sclera was also biopsied, and staining with Ziehl–Neelsen demonstrated AFB scattered in scleral tissue (Figure 3(a)). Histology of the sclera (Figure 3(b)) and the cervical lymph node (Figures 3(c) and 3(d)) revealed granulomatous caseating inflammation, and sputum for GeneXpert became positive confirming the diagnosis of tuberculosis. His HIV screening test was positive, and subsequently coexisting HIV infection was confirmed with Western Blot (CD4 count of 71 cells/mm^3^). Once the diagnosis of TB scleritis was made, he was started on the standard four-drug regimen for TB and continued for one month before the commencement of antiretroviral treatment (HAART). Treatment was continued uninterruptedly with close observation and follow-up. After two months of combined treatment, he showed evidence of healing scleritis (Figure 1(b)) with no further relapses.

## 3. Discussion

Ocular tuberculosis is a rare form of extrapulmonary TB. Any of the extra- or intraocular tissue can get involved [8]. Scleritis is a rare form of ocular TB commonly presenting as anterior nodular scleritis. The exact pathogenesis of TB scleritis is still in debate. Hematogenous spread from the lungs is considered to be the commonest mechanism by which TB affects eyes, but spread via direct local extension is considered to be rare [2, 5, 7]. Diagnosis of TB scleritis is challenging specially in endemic setting and nonspecific tests such as the tuberculin skin tests, and interferon-gamma release assays are widely used and considered useful diagnostic tools. Since these cannot differentiate between the latent form and active disease, the utility of these tests become limited in the endemic areas [7]. Like any other infectious disease, the definitive diagnosis of TB scleritis is to demonstrate mycobacterium TB in scleral samples by culture or detection of acid-fast bacilli (AFB) in smears [2, 7]. Obtaining ocular samples is invasive and is frequently associated with serious complications such as keratitis, cataract, and even permanent blindness [2, 9, 14, 15]. Even if the samples can be obtained, the yield of identification of TB bacilli from ocular tissue is very low [6, 9, 11, 15]. Owing to the lack of pathognomonic clinical features for the diagnosis of TB scleritis, microbiological diagnosis is vital [2, 14]. Also, it is uncertain to conclude whether the ocular involvement is due to infection in situ or whether due to immunological response to infection [2, 5, 8]. In such settings, histologically as well as microbiologically proven TB scleritis in our patient adds valuable information to the literature.

Most cases of ocular TB typically occur without significant systemic disease. In a study carried out in India, concomitant ocular disease was observed in 1.2% of those with pulmonary TB, with 16.2% and 23.2% of the patients having military TB [9]. In many cases, the typical pulmonary lesions are not present in the chest radiograph, especially when coinfected with HIV [9, 10]. In such situations, CT scan is valuable to demonstrate systemic or pulmonary involvement [9] as how it was useful in our case.

The classical histopathologic feature of granulomas characterized by central caseating necrosis with peripheral macrophages or epithelioid cells, lymphocytes, and Langerhan's giant cells are typical of TB in which horseshoe-shaped peripheral arrangement of multiple nuclei is observed [8, 9]. Though it is considered that it is difficult to differentiate primary from rectivated TB from histology, caseating granulomas tend to occur in the latter [8]. Occasionally, central caseation might be absent in TB granulomas even in immunocompetent individuals [8, 9]. Special stains such as Ziehl–Neelson for AFB are more specific tests for a definitive diagnosis of TB [2, 7]. The initial negativity of Mantoux test in our patient is explained by the coinfection with HIV which made the patient immunocompromised. This may be the reason of unusually high number of neutrophils seen within the granulomas of our patient which is not typical of TB. The demonstration of AFB in the sclera as well as the subsequent molecular diagnosis with positive GeneXpert test confirmed the diagnosis supported by the suggestive radiological evidence.

Management of scleritis depends on the underlying aetiology. Considering the strong association of autoimmune pathology, immunosuppressive therapy is widely used when the disease is not well controlled with the topical therapy. This may bring out catastrophic outcomes in the presence of undiagnosed underlying infections such as TB [2]. Once TB is diagnosed, the definitive treatment is with standard anti-TB drugs. Because of the rarity of this form of TB, there is no consensus regarding its management. However, the common practice is to treat ocular TB in the same as for extrapulmonary TB [2, 5, 9]. Since relapses of ocular TB are commonly observed, it may necessitate an extended course of treatment [2, 9]. The Centre for Disease Control (CDC) recommends four-drug treatment with isoniazid, rifampin, pyrazinamide, and ethambutol for a total of 2 months, followed by an additional 4–7 months of dual therapy with isoniazid and rifampicin [2]. Since there are postulations with regard to the immunologically mediated pathophysiology of ocular TB, which is supported by the observation of clinical worsening of the disease with the introduction of ATT, certain literature suggests the combination of steroids with ATT [2, 9]. We treated our patient who was already immune suppressed as a microbiologically proven case of ocular TB with ATT alone which gave satisfactory results.

Current guidelines recommend treatment with antiretroviral therapy in all retroviral-infected patients irrespective of the initial CD4 count [16]. But when it comes to coinfection with tuberculosis considering the possibility of immune reconstitution syndrome, the antiretroviral treatment is preceded by some duration of ATT. This delay of commencement of HAART is decided by the initial CD4 count [16–18]. Our patient is currently on both ATT and HAART with remarkably good outcomes.

## 4. Conclusion

Tuberculous scleritis is a rare form of extrapulmonary TB. The demonstration of AFB in direct smear or with culture is the best way to confirm the diagnosis, and coinfection with HIV should be investigated when such rare presentations are observed. In confirmed cases of TB scleritis, the standard ATT regimen should be commenced without a delay to prevent progression.

## Abbreviations

TB: Tuberculosis
MTB: *Mycobacterium tuberculosis*
AFB: Acid-fast bacilli
CT: Computed tomography
ATT: Antituberculosis treatment
HAART: Highly active antiretroviral therapy.
